# 

**Published:** 1958-04

**Authors:** 


					I
THE
MEDICAL JOURNAL
OF THE
SOUTH-WEST
Incorporating
The Bristol Medico-Chirurgical Journal
April 1958
VOL. 7j (ii). No. 268 Price 4/-

				

